# Alcohol consumption—none is better than a little

**DOI:** 10.1007/s00059-024-05280-z

**Published:** 2024-11-04

**Authors:** Bernhard Maisch

**Affiliations:** 1 Praxisgemeinschaft Marburg, Erlenring Center, Marburg, Germany; 2 Herz- und Gefäßzentrum Marburg (HGZ), Philipps University Marburg, Marburg, Germany; 3 Feldbergstr. 45, 35043 Marburg, Germany

**Keywords:** Intoxicant, Heart, French paradox, Cocaine, Combined misuse, Noxe, Herz, „French paradox“, Kokain, Kombinierter Missbrauch

## Abstract

Alcohol is socially accepted and widely consumed as a recreational beverage. Furthermore, it is used as a disinfectant for medicinal purposes and as a cultural asset it is also part of religious rituals. However, it is also an intoxicant and an addictive substance. The deleterious side of alcohol is reflected in the fact that around 3 million people worldwide die every year as a direct or indirect result of alcohol consumption. For several decades, epidemiological studies suggested that drinking alcohol in moderate quantities was beneficial. This was referred to as the “French paradox,” which described differences in mortality between France and Finland mainly, but also other countries, that were found in epidemiological studies. The difference in the levels of alcohol consumption was found to explain the differences in mortality in view of the otherwise similar risk factors. When alcoholic drinks per day were plotted against all-cause mortality this led to a J-shaped curve. This finding represented a window of benefit for moderate alcohol consumption. However, the recent publication by Zhao et al. in 2023 revisited the relationship between the quantity of alcohol consumed and mortality risk and led to a paradigm change, which has influenced not only the recommendations of *Canada’s Guidance on Alcohol and Health* but also the recommendations and guidelines of major health organizations: “No alcohol is better than a little.” The J‑shaped curve as an explanation of the French paradox became a linear relationship between the amount of alcohol consumption and the increasing mortality from tumors and cardiovascular diseases. The renewed review of several control groups in previous epidemiological studies revealed a recruitment error due to the inclusion of abstinent ex-drinkers. Taking this bias into account, the alcohol-friendly view of small amounts of alcohol being cardioprotective had to be revised. The combined misuse of alcohol and other risk factors for carcinogenesis and heart diseases still needs further attention. The misuse of both alcohol and cocaine led to the conclusion that when the two risky substances are consumed together, it is even more detrimental than the mere sum of the two.

## Abstract (de)

Alkoholika sind gesellschaftlich anerkannte Genuss- und Nahrungsmittel und werden weltweit konsumiert. Alkohol wird als Desinfektions- und Lösungsmittel für medizinische Zwecke verwendet und ist als Kulturgut auch Bestandteil religiöser Rituale. Es ist aber auch ein Rausch- und Suchtmittel. Als deren direkte oder indirekte schädlichen Folgen sterben jedes Jahr etwa 3 Mio. Menschen. Epidemiologische Studien vermittelten über mehrere Jahrzehnte den Eindruck, dass der Konsum von Alkohol in moderaten Mengen einen gesundheitlichen Nutzen hat. Dies wurde als „French paradox“ bezeichnet, das Unterschiede in der Mortalität am Beispiel Frankreich und Finnland, aber auch anderen Ländern beschrieb. Dabei zeigte sich ein Fenster für einen gesundheitlichen Nutzen durch mäßigen Alkoholkonsum im Rahmen eines Vergleichs des täglichen Alkoholkonsums bei ein bis zwei „Drinks“ und dem Mortalitätsrisiko in einer J‑förmigen Kurvenbeziehung. Mit der Veröffentlichung von Zhao et al. 2023 kam es zu einem Paradigmenwechsel, dem sich nicht nur *Canada’s Guidance on Alcohol and Health*, sondern auch die Leitlinien und Empfehlungen anderer internationaler medizinischer Gesellschaften anschlossen. Die J‑Kurve, entwickelt aus dem „French paradox“, wurde zu einer linearen Beziehung zwischen steigendem Alkoholkonsum und dem zunehmenden Mortalitätsrisiko durch Tumor- und Herz-Kreislauf-Erkrankungen. Die erneute Überprüfung mehrerer Kontrollgruppen in früheren Studien ergab einen Rekrutierungsfehler bei Verwendung abstinenter ehemaliger Trinker. Unter Berücksichtigung dieser Tendenz muss die früher alkoholfreundliche Beziehung zwischen kleinen Mengen Alkohol revidiert werden: Kein Alkohol ist besser als Konsum in kleinen Mengen. In Bezug auf den gemeinsamen Missbrauch von Alkohol und anderen Risikosubstanzen für die Krebsentstehung und für kardiovaskuläre Erkrankungen besteht weiterer Klärungs- und Handlungsbedarf. Wie der gemeinsame Konsum von Kokain und Alkohol zeigt, schaden beide riskanten Substanzen zusammen mehr als dies aus der Summe beider zu erwarten wäre.

## Definitions

Alcohol is a socially accepted recreational beverage. As a cultural asset, it is also part of religious rituals. It may be exhilarating when consumed in small volumes, and intoxicating in greater volumes. Alcohol is both an intoxicant and an addictive substance. Its deleterious side is reflected in the fact that around 3 million people worldwide die every year as a direct or indirect result of alcohol consumption. It is an addictive substance, when individuals depend on its regular use: Alcohol dependence is then classified as a disease according to the *International Classification of Disease, 10th Revision* (ICD-10; [1]). At least three out of six criteria have to be fulfilled for the diagnosis of alcohol dependence:

1. Craving (a strong desire or sense of compulsion to consume the psychoactive substance alcohol)
2. Loss of control over intake in terms of its onset, termination, or levels of use
3. A withdrawal state when the use of the substance has ceased or has been reduced (withdrawal syndrome)
4. Evidence of tolerance, such that increased doses of alcohol are needed to achieve the previously achieved desired effects
5. Progressive neglect of other interests and pleasures due to substance abuse
6. Persistent substance use despite evidence of harmful mental and/or physical consequences

Alcohol is also a noxious factor, with around 3 million people worldwide dying every year as a result of its consumption.

In 1960 Elvin M. Jellinek introduced a classification of five types of alcohol-dependent drinkers, which has become obsolete but is still in common use. The five types of drinkers are named after the first five letters of the Greek alphabet [2]:

- *Alpha drinkers* drink when problems arise. There is no physical dependence or loss of control but there is psychological dependence.
- *Beta drinkers *drink on certain occasions conforming with social patterns of consumption. There is danger of addiction.
- *Gamma drinkers *are psychologically addicted to drinking.
- *Delta drinkers *are level drinkers with physical dependence. They need a constant level of alcohol in their blood to function in everyday life. Under this threshold, they suffer from withdrawal symptoms.
- *Epsilon drinkers *are interval drinkers with psychological dependence. Periods of abstinence are followed by episodes of excessive consumption with loss of control and memory.

In Germany the habit of episodic drinking of large volumes of alcohol (five or more drinks per day) by young men has fortunately decreased from 47.5% to 33.6% and in women from 19.8% to 16.7% in the period from 1995 to 2021.

## A historic paradigm change

A dramatic paradigm change in the public perception of the risk of alcohol consumption even in smaller amounts began on 4 April 2023, when the *New York Times* reported on a reappraisal of major epidemiological studies on alcohol consumption and the risk of all-cause mortality [3] from two landmark publications by Zhao et al. [4] and by Paradis et al. [5] for the Canadian Center on Substance Use and Addiction. *Canada’s Guidance on Alcohol and Health 2023* consequently marked a historic turnaround not only in terms of the risk assessment for tumors and for health in general but also for cardiovascular prevention. *HERZ/Cardiovascular Diseases *reported early on this turnaround for health and for heart diseases [6]. In order to better understand the paradigm change, which will be discussed in detail here, some basic aspects pertaining to alcohol and its interference in cells, organs, and the body will set the appropriate stage.

## How does alcohol affect human cells, organs, and the entire body?

Alcohol is a cell poison that after consumption is distributed throughout the body via the blood. It also crosses the blood–brain barrier, which is a common experience at different states of social behavior and consciousness. It damages organs, nerve cells, and the immune system. In addition, alcohol consumption promotes the development of various types of cancer: In Germany around 4% of all new cancer cases are a result of alcohol consumption. Its detrimental effect is differentially expressed: Even small doses can cause cancer, while higher doses cause hypertension, arrythmia, and heart failure.

After consumption, alcohol (C_2_H_5_OH) is degraded by alcohol dehydrogenase (ADH) to acetaldehyde (C_2_H_4_O), which is cytotoxic to cells and organs in various ways and to different extents [7, 8]. Alcohol exerts oxidative stress in cells, enhances inflammation, and influences the regulation of estrogen. Thus, different mechanisms can contribute to carcinogenesis. Major target organs are the oral cavity, throat, larynx, esophagus, colon, rectum, liver, and mammary glands.

In 2022, newly discovered carcinomas and tumors affected 493,200 patients. The number of male and female patients who died in 2022 and were chronic drinkers can be derived from Table 1; except for the case of breast cancer, the male gender is predominant. Obviously, emerging bronchial carcinoma at 29,138 cases and prostate carcinoma at 15,590 cases are not listed, as they were considered causatively unrelated to chronic alcoholism.

**Table 1:** Mortality data for male and female cancer patients associated with chronic alcohol consumption in 2022 in Germany (modified from [9] and [6])

Type of tumor	ICD-10 code	Male (*n*)	Female (*n*)
Colon and rectum	C18–20	8050	340
Breast (mammary glands)	C50	0	800
Oral cavity, throat, and larynx	C01–06, C09–10, C12–14	2830	200
Liver	C22	1610	640
Esophagus	C15	930	140
Larynx	C32	570	20

ICD-10 code of diseases, *International Statistical Classification of Diseases and Related Health Problems 10th Revision* [1]

The International Agency for Research on Cancer (IARC) and the *Alkoholatlas Deutschland 2022 *based the figures in Table 1 on reports from health services for individuals who had consumed more than two or three standard drinks per day for male patients and more than one or two drinks for female patients. The definition of one standard drink with reference to selected beverages is presented in Table 2 [6].

**Table 2:** What corresponds to a “standard drink”? (modified from [6])

Beverage	Alcohol in %	Volume in mL	Mass in oz^a^
Beer	5	341	12
Cider	5	341	12
Wine	12	142	5
Liquor	40	43	1.5

^a^1 ounce (oz) = 28.35 g = 28.42 mL

## Alcohol consumption in Germany

About 10% of Germans consume alcohol daily in amounts that are likely to be a risk to health: This corresponds to 7.9 million inhabitants who drink one to two beers daily or regularly (Table 2).

On average, German citizens consumed 105.9 L beer, 20.5 L wine, 5.4 L liquor, and 3.7 L champagne per year and per capita.

Men drink predominantly beer, women, wine. Beer consumption in Germany, with 5.6 L pure (100%) alcohol consumed per year, places it in fourth place in European ranking [9]. Remarkably, from 1995 to 2021 the weekly beer consumption in men between 18 and 59 years of age decreased from 3.5 to 2.1 L, and in women from 1.2 to 0.8 L. Wine and champagne consumption has not changed much and remains at 0.5 L and 0.4 L, respectively, per person per week. The consumption of liquor in men still lies at 0.08 L, in women at 0.04 L, per week and per person.

## Alcohol-associated carcinogenesis, morbidity, and mortality

Whereas Table 1 presents the figures for tumors recorded in men and women in Germany in 2022 [6], Table 3 focuses on diseases exclusively and directly cause by alcohol (second column). The third column refers to symptoms and diseases in which alcohol consumption leads to an increased risk of morbidity.

**Table 3:** Diseases caused directly by alcohol consumption or associated with an increased risk of morbidity through alcohol consumption (modified from [6] and [9])

	Alcohol as exclusive cause	Increased morbidity risk through alcohol consumption
Psyche	Addiction, behavioral disorders, withdrawal syndrome	Cofactor for various psychopathological processes
Nervous system	Degeneration, polyneuropathy	Alzheimer disease, depression, epileptic seizures
Abdominal organs	Gastritis, hepatitis, fatty liver and liver cirrhosis, pancreatitis	Carcinogenesis of abdominal organs: see Table 1
Heart and vessels	Alcoholic cardiomyopathy	Hypertension, heart failure, stroke, coronary artery disease (?)
Lung and breast	Breast cancer	–
Pregnancy	Alcoholic embryopathy	–
Immune system and infection	Negative modulation of the inflammasome and of autoimmune diseases	Myocarditis and pericarditis, tuberculosis, HIV/AIDS, pneumonia
Metabolism	–	Type 2 diabetes
Other causes	Pseudo-Cushing, niacin deficiency (pellagra), intoxications, myopathies	Accidents, lesions, atrocities and acts of violence

In most conditions that are listed in Table 3, the probability of falling ill or dying increases with the amount of alcohol intake. This applies in particular to carcinogenesis. Consequently, only by drinking no alcohol at all can all health risks be avoided.

## Alcohol and the heart

### Early descriptions

Historically, alcohol and heart diseases first received attention in 1873, when W.H. Walshe described focal myocardial cirrhosis in alcohol-dependent patients [10]. Wilhelm Münzinger published “Das Tübinger Herz, ein Beitrag zur Lehre von der Überanstrengung des Herzens” (*The Tübingen Heart, A Contribution to the Doctrine of Cardiac Overexertion*) 4 years later. It was a result of chronic wine consumption with fibrotic changes and myofiber hypertrophy with an concomitant arsenic intoxication [11].

**Fig. 1:**
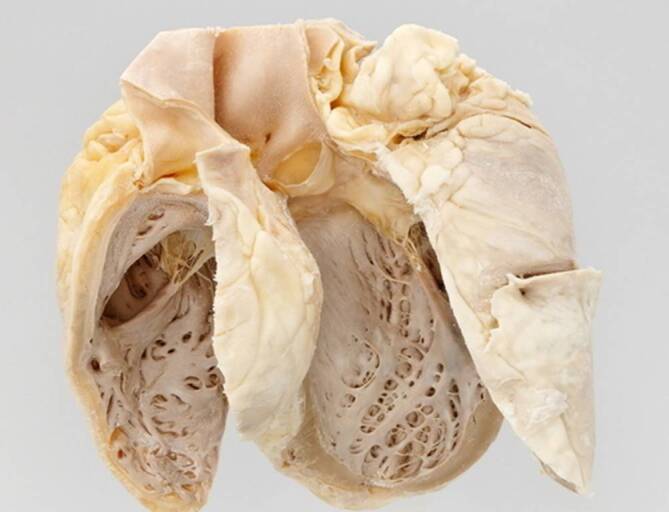
“Münchener Bierherz”: Munich beer heart. (© Philipp Mansmann in [13])

In 1884 the “Munich beer heart” was described by Otto von Bollinger, which combined adipose tissue, fibrosis, and hypertrophic myofibers (Fig. 1; [12]).

When reporting in 1887 on two patients with severe alcohol consumption who benefitted from abstinence, Maguire suggested that alcohol itself was poisoning the heart. Strümpell and later William Osler in 1892 in his textbook *Principles and Practices of Medicine* listed alcoholism as a cause of cardiac dilatation and hypertrophy. Graham Steell In 1893 reported on 25 cases with chronic alcoholism causing “muscle failure of the heart” [14]. In his 1906 textbook *The Study of the Pulse*, William MacKenzie first used the term “alcoholic heart disease” [15]. In his review article in 1972, Bridgen introduced the term “alcoholic cardiomyopathy” [16].

Currently, all of the aforementioned historical publications fit nicely into today’s definition of alcoholic cardiomyopathy [17]. Their overt clinical phenotype was heart failure as a consequence of low output.

### Beriberi cardiomyopathy—a different form of heart failure

In 1930, C.S. Keefer described, under the term “beriberi heart disease,” the consequence of thiamine (vitamin B_1_) deficiency in chronic alcohol-dependent, malnourished individuals as high-output heart failure [18]. High-output failure is by definition cardiac output of >8 L/min or a cardiac index of >3.9 L/min/m [19, 20]. The concept of “beriberi heart disease” dominated thinking about alcohol and the heart for decades and caused many to doubt that alcohol was the true cardiotoxic substance [21].

### Quebec beer-drinkers’ cardiomyopathy

Bonenfant et al. in 1967 [22] described the pathologic–anatomic features of cobalt beer-drinkers’ cardiomyopathy, while Morin and Daniel [23] explained the underlying etiology. Quebec’s beer brewers, as well as some in the United States, had used cobalt as foam stabilizer. Cobalt is an essential trace element in the form of cobalamin in vitamin B_12_, but it is toxic in the doses used in Quebec. Cobalt toxicity to the heart received renewed interest in a case in which the cobalt intoxication resulted from a defective hip prothesis [24].

It was Regan in 1971 who described the damage to the heart and cardiac function in acute and chronic alcohol consumption [25]. This consumption proved to have negative inotropic effects as assessed by his “contractility index.” Conduction disturbances in chronic alcohol abuse and arrhythmias in acute excessive alcohol abuse also received attention [26, 27]. In 1978 Ettinger et al. coined the term “holiday heart” when describing the remarkable increase in patients who were admitted to New York’s hospitals with arrhythmias during the Christmas holidays after social consumption of various forms of alcohol [27].

### Alcohol and hypertension

Whereas initial vasodilatation may lead to a small decrease in blood pressure after acute alcohol consumption, one expects in chronic use of larger volumes, e.g., several liters of beer per day, hypertension that may be explained by the volume overload [6, 17, 28, 29].

## The French paradox

### The French paradox and saturated fat consumption

**Fig. 2:**
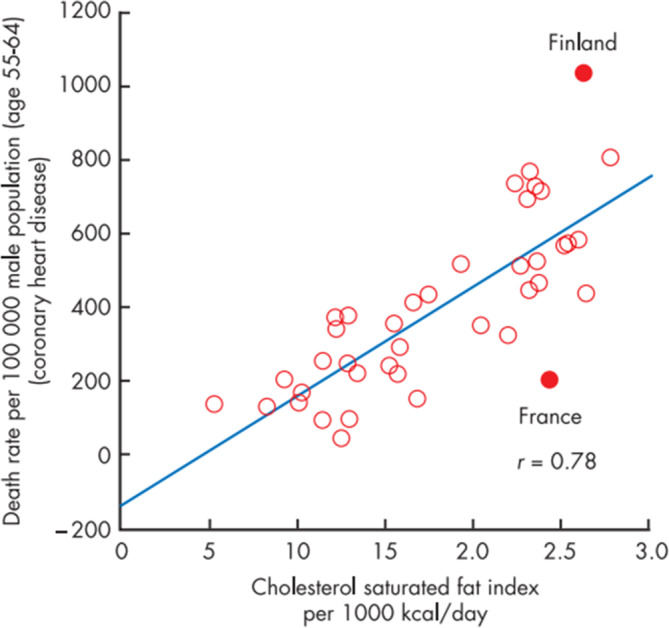
Correlation of the mortality rate from coronary heart disease (1977) with daily dietary intake (from 1976 to 1978) of cholesterol and saturated fat as expressed by the cholesterol–saturated fat index per 1000 kcal in 40 countries. Note the difference between Finland and France. (With permission from [31])

The French paradox relates to the surprising observation that the mortality from coronary heart diseases (CHD) on a logarithmic scale in France was much lower than in the majority of other countries, when compared with the dietary intake of cholesterol (Fig. 2; [30]). The initial explanation for the low CHD death rates despite a high intake of dietary cholesterol and saturated fat was formulated by French epidemiologists in the late 1970s and early 1980s [31]. France and Finland were countries that had similar intakes of cholesterol and saturated fat, but consumption of vegetables and vegetable oil containing monounsaturated and polyunsaturated fatty acids was greater in France than in Finland. At that time France was and continues to be a country with a low incidence and mortality from CHD. After having defined a cholesterol–saturated fat index (CSI), Artaud-Wild et al. [30] studied the relation between the CSI and CHD mortality (per 100,000 men aged 55–64 years) for 40 countries. France had a CSI of 24 per 1000 kcal and a CHD mortality rate of 198, whereas Finland had a CSI of 26 per 1000 kcal and a CHD mortality rate of 1031 (Fig. 2).

### The French paradox and wine consumption

St Leger et al. [32] postulated in 1979 that the cardioprotective effect in France may come from moderate but regular consumption of alcohol. Several publications followed this line of reasoning [17, 33–35]. Discussions focused on different drinking habits and on red wine in particular. Renaud and De Lorgeril [31] compared age-standardized annual mortality from CHD and related risk factors in the populations from the MONICA project, including data from French centers. The mean serum total cholesterol concentrations were similar in France, in the United States, and in the United Kingdom. From the regression analysis between death rate from CHD and consumption of dairy fat and wine, the authors concluded that the French paradox may be caused by a high consumption of wine. Their analysis was the beginning of a series of studies analyzing the relation between wine and CHD and of various mediators of vasodilatation and cardioprotection, which were in fact components of wine, but of red wine in particular. Promising candidates were phenols, e.g., resveratrol, quercetins, and other flavonoids. Thrombocyte aggregation inhibition was found to be increased, as was high-density lipoprotein (HDL; [33, 34]).

**Fig. 3:**
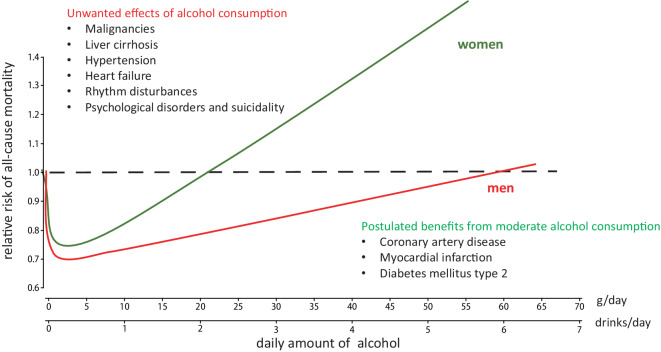
Alcohol consumption and mortality. Alcohol consumption in women (green) and men (red) showing a J-shaped curve compared with the relative risk of total mortality in Europe. (Data from Di Casteluovo A et al. 2006 [33] were implemented in a schematic graph from Flesch et al. 2016 [34])

The epidemiological description resulted in the J‑shaped curve. The dip was reached with moderate alcohol consumption of 3–5 g alcohol per day, corresponding to one drink per day in women and one or two drinks per day in men, as described by Di Casteluovo et al. in 2006 [33]. Figure 3 compares alcohol consumption in women and men in Europe [34].

In their updated meta-analysis, Di Casteluovo et al. [33] concluded that there may be “the existence of potential windows of alcohol intake that may confer a net beneficial effect of drinking, at least in terms of survival, both in men and in women. Heavy drinkers should be urged to cut their consumption, but people who already regularly consume low to moderate amounts of alcohol should be encouraged to continue.”

**Fig. 4:**
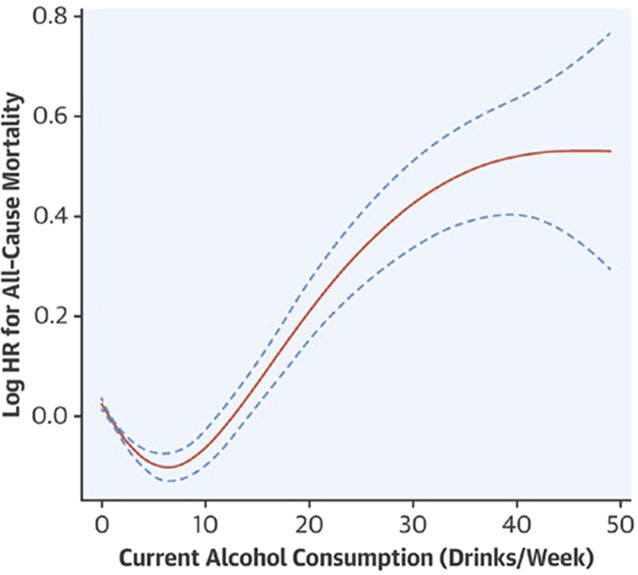
Alcohol consumption and all-cause mortality risk in US adults. HR hazard risk. (With permission from [35])

In 2017 Xi et al. [35] compared the hazard ratio for all-cause mortality in a logarithmic scale with the number of drinks per week in US adults and also found a J-shaped curve (Fig. 4).

Their analysis included 333,247 participants aged ≥18 years who were part of a survey from 1997 to 2009. The mean follow-up was 8.2 years. Overall, 34,754 participants had died, 8947 from cardiovascular causes and 8427 from cancer. The reference group comprised lifetime alcohol abstainers, in order to avoid the bias that former alcoholics were part of the control group thus confounding the results by an “abstainer bias.” The argument that abstainers and ex-alcoholics might have been sicker beforehand (“sick quitter phenomenon”) was counteracted by looking at the hospital files of the non-alcoholic controls and excluding those from the control group. This second model computation, too, showed a J-shaped curve with a window for low or moderate alcohol consumption. The authors concluded that “light and moderate alcohol intake might have a protective effect on all-cause and CVD-specific mortality in US adults.”

## Canada’s guidelines for alcohol and health—a paradigm change

The two landmark publications by Zhao et al. [4] and by Paradis et al. [5] for the Canadian Center on Substance Use and Addiction marked the turnaround not only in the risk assessment for tumors and for health in general but also for cardiovascular prevention. In 2023 Zhao et al. [4] re-examined 107 cohort studies with 4.8 million participants. The essential component of their reassessment was the question of whether a daily alcohol consumption of less than 25 g would lead to a reduction in all-cause mortality. They corrected for a misclassification by ex-alcoholics in 86 out of 107 studies. This led to a readjustment, since in the group of ex-alcoholics the mortality risk was significantly higher than in the remaining reference group of permanent non-alcoholics.

When the authors used as another potential reference group individuals with occasional alcohol consumption, only a non-significant cardioprotective benefit was observed.

**Fig. 5:**
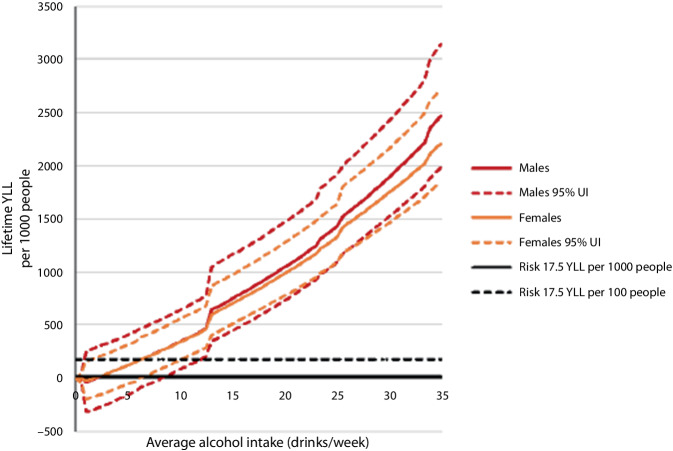
Depicts years of lifetime lost (YLL) attributable to alcohol use at varying levels of average alcohol intake. (From [5], reproduced with permission from the Canadian Centre on Substance Use and Addiction)

Remarkably, in women the mortality risk related to alcohol consumption of 25 g/day was considerably higher than in men. Figure 5 depicts an almost linear relationship between years of life lost (YLL) attributable to alcohol use at the varying levels of average alcohol intake [5].

Under the assumption of a baseline threshold of 17.5 YLL per 1000 people by alcohol consumption (solid black horizontal line at 0 in Fig. 5) the innoxious amount of alcohol is two standard drinks for men and women per week.

Under the assumption of a baseline threshold of 17.5 YLL per 100 people by alcohol consumption (dashed black horizontal line above 0 in Fig. 5) the harmless amount of alcohol is six standard drinks per week for men and women alike. The threshold of 17.5 was defined according to the criteria of the Institute for Health Metrics and Evaluation (2021; [36]).

**Fig. 6:**
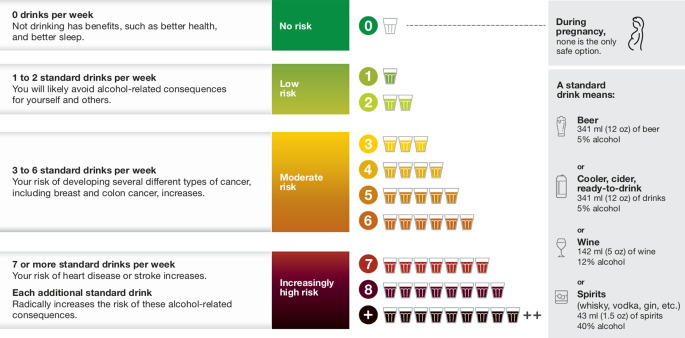
Mortality risk by different volumes of alcohol per week. (From [5], reproduced with permission from the Canadian Centre on Substance Use and Addiction)

These findings have influenced not only Canadian but also American and European guidelines. The recommendations of the Canadian Center on Substance Use and Addiction (Fig. 6) are clear: no risk with zero alcohol, low risk with one or two standard drinks per week. Drinking more alcohol endangers health and prognosis due to an increased risk of carcinogenesis and cardiovascular diseases.

The underlying pathophysiological causes have been summarized in [5] and [6] and are the following:

- Increased infection rate of the respiratory tract by alcohol [37].
- Even small amounts of alcohol show no reduction in the risk of carcinogenesis [38–41].
- There is no threshold for the risk of hypertension [42].
- The risk for cerebral stroke is slightly reduced for embolic forms in moderate alcohol consumption, but is substantially increased in the case of subarachnoid hemorrhage; overall, the results are ambivalent [43].
- Recent studies have also questioned the protective effect of moderate alcohol consumption [44].
- Special attention has been paid to binge drinking [45, 46] and to genetic predisposition [47].

## Alcohol and other toxic agents

The combination of alcohol consumption with other risky substances such as smoking, vaping, and non-medical drug abuse or drugs such as heroine, marijuana, cannabinoids, cocaine, crystal meth (methamphetamines), and other addictive substances deserves continuous attention because little is known on the synergistic but negative health effects. One example out of many is alcohol and cocaine.

### Cocaine

Cocaine is a tropane alkaloid compound that can be extracted from the leaves of the Andean shrub *Erythroxylon coca*. It was originally used during local surgeries as an anesthetic agent in the 1880s before it became a recreational drug in the 1970s. In the 1980s its use in the United States reached the size of an epidemic with an estimated 5.8 million users in the year 1985 [48]. In 2016, the total number of cocaine users was estimated to be 18.2 million worldwide [49]. At that time, approximately 34% of the cocaine users resided in North America, and 20% resided in Western and Central Europe. Young adults aged 18–25 were the most common cocaine users (1.4%).

**Fig. 7:**
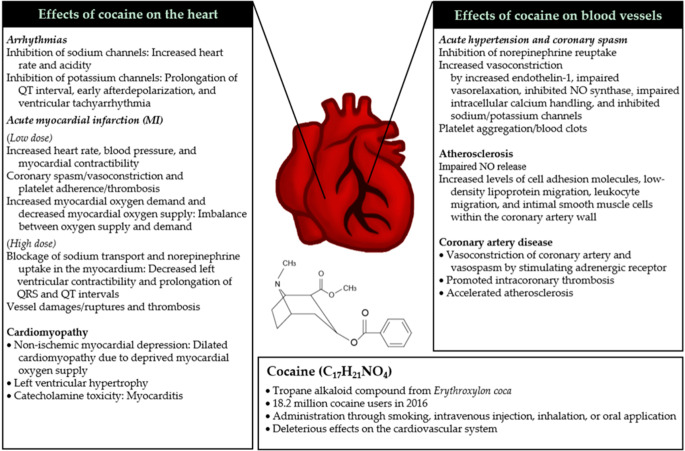
Effects of cocaine on cardiovascular health. The use of cocaine (bottom) results in both acute (italic) and chronic (normal) changes in the heart (left) and blood vessels (right). NO nitric oxide. (From [52] © 2019 by the authors. Licensee MDPI, Basel, Switzerland. This article is an open access article distributed under the terms and conditions of the Creative Commons Attribution (CC BY) license: http://creativecommons.org/licenses/by/4.0/)

Cocaine stimulates the sympathetic nervous system by inhibiting the reuptake of norepinephrine, dopamine, and serotonin through interactions with each transporter. This leads to an exaggerated, prolonged sympathetic nervous system activity [50]. Cocaine also blocks sodium/potassium channels, which induces abnormal, depressed cardiovascular profiles [51]. The abuse of cocaine is associated with an increased risk of subsequent cardiovascular complications such as hypertension, coronary spasm, arrhythmias, myocardial infarction, cardiomyopathy, atherosclerosis, and coronary artery disease (Fig. 7; [52]).

## Cocaine and alcohol

Cocaine in combination with alcohol creates new metabolites such as cocaethylene. Their concurrent abuse is more than “the sum of two parts” [53]. Cocaethylene in turn significantly increases cocaine levels in the blood. Their combined effects are therefore stronger than either cocaine or alcohol alone or both added up. This leads to increased toxicity to the heart, liver, and other major organs. The metabolites also remain in the body for a longer time than cocaine alone; thus, their toxic effects last longer. Alcohol slows the removal of another metabolite, ethylbenzoylecgonine, from the kidneys. This, in turn, raises the blood levels of cocaine and cocaethylene [54].

Presented here is an interview with a cocaine- and alcohol-dependent 49-year-old male individual from an urban drug district in Lübeck, a hub for drug-trafficking. The name of the person has been changed and is referred to as “WZ.” The interview was conducted by Philipp Maisch when working as a nurse. Consent for the use of this interview was obtained from WZ:

- **PM:** How old are you and how long have you been on drugs?
  **WZ:** I am a 49-year-old German on drugs, particularly cocaine (“koka”) and alcohol in irregular fashion since the age of 20.
- **PM:** What drugs have you taken and how did you use them?
  **WZ:** I have tried several drugs but at the end I always landed up using koka. I am unable to stop its use, although I am aware that I will die from it. I started with nasal inhalation, changed to i.v. injections after 2 years; presently I inhale it as a free base.
- **PM:** Do you also consume other drugs and alcohol?
  **WZ:** At the beginning I also consumed marijuana and several other drugs. I have the impression that alcohol prolongs the effect of cocaine.
- **PM:** What are your physical complaints?
  **WZ:** Through consumption of stretched drugs I suffered from pulmonary emboli and obstipation. I had to undergo surgery for embolectomy where they found even small particles of plastic. I also had my first coronary stent 12 years ago and received my first permanent pacemaker.
- **PM:** Your first pacemaker?
  **WZ:** I fainted a second time, when I consulted my physician. I was taken to hospital and they changed the battery of the pacemaker.
- **PM:** Has this changed your drug consumption?
  **WZ:** Immediately after surgery I left the hospital to get cocaine, for which I longed desperately. I feel totally incomplete without it. But I have changed from i.v. to smoking base (“crack”) alone.
- **PM:** You had a lot of problems with the mixed consumption of cocaine and other drugs. Did this change your habit?
  **WZ:** Of course, the doctors advised me more than once to quit, but I cannot live without crack.
- **PM:** Being aware of your current situation, what has gone wrong from the beginning?
  **WZ:** I don’t care. Because after the first consumption, the only desire is to have another one. This is a permanent dependence for more than 26 years.

### Increased risk of stroke

Sudden stroke is a possible complication when using both cocaine and alcohol. Cocaine increases the risk of stroke by vasospasm, raising the heart rate and blood pressure. In this way it can cause sudden cerebral hemorrhage or increase the risk of thrombotic sequelae. Cocaethylene adds to the risk of stroke even more because it can remain in the body for days to weeks. It can increase cravings for cocaine.

Both cocaine and cocaethylene raise levels of the brain chemicals dopamine and serotonin and are likely to block their reuptake. This increases the stimulant effects on the body, which can lead to impulsive and violent behavior, panic attacks, anxiety, and depression.

### Increased risk of heart-related problems

The rise of cocaethylene and cocaine in the combined consumption of alcohol with cocaine increases liver and heart toxicity. The greatest dangers are sudden heart-related problems, such as a heart attack or arrhythmias as described in detail in Fig. 7, and are more frequent than by cocaine consumption alone. Further long-term longitudinal studies to explore the extent of consuming more than alcohol—e.g., in combination with cocaine, crystal meth, or recreational amounts of marijuana—are mandatory.

## Conclusion

- The risk of mortality increases with the amount of alcohol consumption. Only by abstaining from alcohol can no additional risk be expected.
- The risk remains low with two standard drinks per week. It remains moderate with three to six standard drinks per week. But there is no benefit with moderate consumption. This is in fact a paradigm change.
- If more than two standard drinks are consumed on one occasion, there is an additional risk for increased violence, particularly by men.
- Abstaining from alcohol is a longstanding recommendation for pregnant women and also breastfeeding mothers. There is no threshold for alcohol in unborn or newborn children, when breastfeeding is practiced. Abstention from alcohol, narcotics, and smoking also applies for pregnant or breastfeeding women.
- Violence with fatal outcome is encountered in uncontrolled binge drinking mostly by men.
- The combined use of alcohol and drugs as exemplified by parallel consumption of alcohol and cocaine increases health and cardiovascular risk by more than the sum of the two drugs.
- For all drugs, although recreational use may be legally allowed, it should be clearly kept in mind that “none is better than a little.”

## Declarations

For this article no studies with human participants or animals were performed by any of the authors. All studies mentioned were in accordance with the ethical standards indicated in each case.
